# The effect of concurrent elevation in CO_2_ and temperature on the growth, photosynthesis, and yield of potato crops

**DOI:** 10.1371/journal.pone.0241081

**Published:** 2020-10-21

**Authors:** Yun-Ho Lee, Wan-Gyu Sang, Jae-Kyeong Baek, Jun-Hwan Kim, Pyeong Shin, Myung-Chul Seo, Jung-Il Cho

**Affiliations:** Crop Production and Physiology Division, National Institute of Crop Science, Rural Development Administration, Wanju, Jeollabuk-do, Republic of Korea; Kyung Hee Univeristy, REPUBLIC OF KOREA

## Abstract

Global climate change accompanied by continuous increases in atmospheric carbon dioxide (CO_2_) concentration and temperature affects the growth and yield of important crops. The present study investigated the effect of elevated temperature and CO_2_ concentrations on the growth, yield, and photosynthesis of potato (*Solanum tuberosum* L. cv. Superior) crops using Korean Soil-Plant-Atmosphere-Research chambers that allow the regulation of temperature and CO_2_ concentration under daylight conditions. Based on the average temperature from 1991 to 2010 in the Jeonju area, South Korea, potato plants were exposed to four different conditions: ambient weather (400 μmol mol^-1^, aCaT), elevated temperature (+4°C, aCeT), elevated CO_2_ concentration (800 μmol mol^-1^, eCaT), and concurrently elevated CO_2_ concentration and temperature (eCeT). Under aCeT conditions, the temperature exceeded the optimal growth temperature range towards the late growth phase that decreased stomatal conductance and canopy net photosynthetic rate and subsequently reduced biomass and tuber yield. Stomatal conductance and chlorophyll concentration were lower under eCaT conditions than under aCaT conditions, whereas late-growth phase biomass and tuber yield were greater. Compared to other conditions, eCeT yielded a distinct increase in growth and development and canopy net photosynthetic rate during tuber initiation and bulking. Consequently, biomass and canopy net photosynthesis increased, and tuber yield increased by 20.3%, which could be attributed to the increased tuber size, rather than increased tuber number. Elevated CO_2_ reduced chlorophyll, magnesium, and phosphorus concentrations; reducing nitrogen concentration (by approximately 39.7%) increased the C:N ratio. The data indicate that future climate conditions will likely change nutrient concentration and quality of crops. The present study shows that while elevated temperature may negatively influence the growth and yield of potato crops, especially towards the late-growth phase, the concurrent and appropriate elevation of CO_2_ and temperature could promote balanced development of source and sink organs and positively effect potato productivity and quality.

## Introduction

Global climate change has accelerated since the beginning of the 21^st^ century. For example, the atmospheric CO_2_ concentration in 1880 was 280 μmol mol^-1^ but it has increased continuously since the industrial revolution and is currently at least 400 μmol mol^-1^. The average annual temperature has increased by 0.8°C since 1880 [1]. Based on the Representative Concentration Pathway (RCP) Scenario 8.5 of the Intergovernmental Panel on Climate Change [2], atmospheric temperature could rise by as much as 4.8°C by around 2100 and the CO_2_ concentration could reach 940 μmol mol^-1^. These predicted increases in atmospheric CO_2_ concentration and temperature are anticipated to affect crop productivity and quality [3–5]. However, each crop variety exhibits different response patterns to changes in temperature and CO_2_ concentration [6–8].

Potato (*Solanum tuberosum* L.) is one of the five major global food crops along with rice, wheat, corn, and soybean [9]. It is important to analyze the effect of climate change on the production of potato plants to secure the future of the global food supply. Modern potato cultivars generally grow well under moderate temperatures around 20°C, and the optimum temperature for the growth of above-ground parts, such as leaves and stems, and the initiation and bulking of the underground tubers are known to be 20–25°C and 15–20°C, respectively [10–13]. The rise in temperature affects almost all biological processes of potato plants including photosynthesis, respiration, and enzyme activities [14, 15]. Long-term exposure to high temperatures has been reported to increase photorespiration rather than photosynthetic rate [16] and has been reported to reduce photosystem II (PS II) activity, chlorophyll concentration, and enzyme activities [10, 17–19]. Multiple studies have reported that the rise in temperature above the optimum level decreased the leaf area and biomass, delayed tuber initiation and decreased the tuber yield through the inhibition of carbon assimilation and its subsequent translocation to tuber [20–24].

In contrast, the increase in atmospheric CO_2_ concentration is generally known to exert a positive influence on crop photosynthesis, growth, and yield [8, 25–29]; potato has been reported to exhibit high yields under high CO_2_ concentrations. The increased yield can be attributed to the increase in the rate of foliar photosynthesis and the facilitated allocation of assimilates to tubers [30]. Recent studies have reported that if CO_2_ concentration increases by two-fold, the biomass and tuber yield of potato plants could increase by as much as 49% [30–33]; some studies have reported that increases in atmospheric CO_2_ levels could also improve water and nitrogen use efficiency [34–36].

Studies have also reported that elevated CO_2_ levels could lead to a decrease in stomatal conductance and chlorophyll concentrations [31, 37–42], as well as to changes in essential mineral concentrations and carbon to nitrogen (C:N) ratios in plants [43–47]. Upon exposure to elevated CO_2_ concentrations for a long period, the carboxylase activity of ribulose-1,5-bisphosphate carboxylase/oxygenase (Rubisco) decreases while the C:N ratio increases due to the high carbon status [36, 48]. Such an increase in the C:N ratio with lower nitrogen levels leads to lower protein content, and it eventually deteriorates the quality of the crop.

Carbon dioxide and temperature are important factors in the growth and development of potato crops. The recent global climate changes have affected significantly the growth, yield, and photosynthesis of the crop. These two factors together influence the growth and photosynthesis of the crop, not independent of each other [49]. It is important to investigate how crops are affected by elevated temperature and CO_2_ concentration, separately and in combination. Most studies on the effect of elevated temperature or CO_2_ concentrations have been conducted using closed-top fields (CTFs), open-top chambers (OTCs), or free-air carbon dioxide enrichment (FACE) [26, 47, 50–52] and have generally neglected to investigate the effect of concurrent elevations in CO_2_ concentration and temperature. The SPAR facility is known to be optimized equipment to analyze the growth and development of crops at the canopy-level under precisely controlled environmental conditions that can control temperature and CO_2_ concentration under daylight conditions [53]. Using the Korean Soil-Plant-Atmosphere-Research (KSPAR) chambers, the present study aimed to investigate the effect of long-term exposure to elevated CO_2_ concentration and temperature on potato plants, with the goals of identifying interactions between i) growth, development, and yield; ii) photosynthetic responses; and iii) the concurrent elevation of CO_2_ concentration and temperature.

## Materials and methods

### Experimental site and Korean Soil-Plant-Atmosphere-Research (KSPAR) chamber

**Fig 1:**
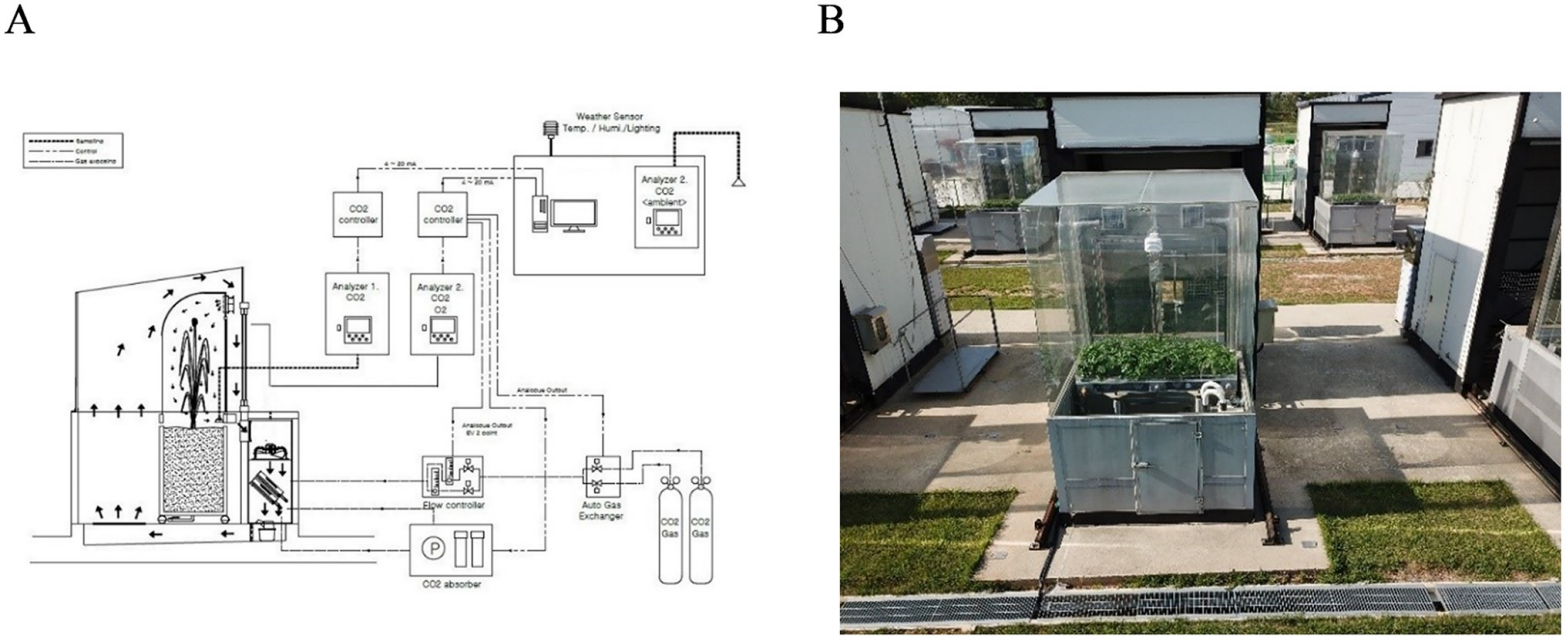
Korean Soil-Plant-Atmosphere-Research (KSPAR) chamber. (A) Schematic diagram, (B) Experimental site in Wanju-gun, Jeollabuk-do, Korea.

The present study was conducted at the National Institute of Crop Science, Wanju, Jeollabuk-do, Korea (35°84' 34" N, 127°04' 84" E). The Korean Soil-Plant-Atmosphere-Research (KSPAR) chamber was a slightly modified version of the SPAR chamber of the USA Agricultural Research Service (ARS) [53]. The most significant feature of the equipment is the double chamber composed of inner and outer chambers and designed to reduce CO_2_ leakage and temperature rise due to radiant-heat generation (Fig 1).

The CO_2_ exchange rate can be measured without the use of N_2_O gas [54]. The inner chamber consists of a structure covered by 10 mm thick Plexiglas (Evonik Industries, Essen, Germany) measuring 2.00 m × 0.95 m × 2.25 m (length × width × height). The lower part of the inner chamber contains a steel soil bin measuring 2.00 m × 0.78 m × 1.42 m (length × width × height). The upper part of the outer chamber is a 4 mm thick clear laminated glass measuring 2.30 m × 2.64 m × 2.50 m (length × width × height) with a light transmissivity of approximately 93–95%.

The inner chamber includes an air-temperature and relative-moisture sensor (TRH-300, Rixen Technology, Taipei, Taiwan) that collects data at 30-sec intervals. The soil bin has a soil temperature and moisture sensor (Drill & Drop, Sentkek Technologies, Stepney, Australia) that collects data at 1-h intervals (CEM20, Thermo Fisher Scientific Inc., Scoresby, Australia). Photosynthetically active radiation was measured using a quantum sensor (SQ-326, Apogee Instruments Inc., UT, USA) at 10-min intervals.

The CO_2_ supply to the SPAR chamber was maintained at a steady level using a gas-pressure sensor (Model 200 IM, Seowon, Yongin, Korea) and a flowmeter. All chambers were equipped with two non-dispersive infrared (NDIR) CO_2_ analyzers (LI-820, LI-COR Biosciences Inc., NE, USA) to monitor the concentration of CO_2_ in the air flowing in and out of the inner chamber. Before the experiment, all CO_2_ analyzers were calibrated for the zero and span values, according to the instructions given in the LI-820 manual.

### Experimental design

Before emergence, the day and night air temperatures were maintained at 20 and 15°C, respectively, and the CO_2_ concentration was maintained at 400 μmol mol^-1^. Starting temperature and CO_2_ concentration was based on the ambient climate conditions or the projected 2100 climate (RCP Scenario 8.5), depending on the treatment [2].

During the experiment, the CO_2_ concentration was maintained at either 400 or 800 μmol mol^-1^; the temperature was maintained at either the daily mean temperature of the Jeonju area (1991–2010) or 4°C warmer. The treatments involved the following conditions: ambient CO_2_ and ambient temperature (aCaT), ambient CO_2_ and elevated temperature (aCeT), elevated CO_2_ and ambient temperature (eCaT), and concurrently elevated CO_2_ and temperature (eCeT). All treatment conditions were arranged in completely randomized design [28].

During the day (05:00–19:00), the CO_2_ concentrations for the two ambient and two elevated CO_2_ conditions were maintained at 400 μmol mol^-1^ and 800 μmol mol^-1^, respectively. The CO_2_ concentration during the night (19:00–05:00) was left uncontrolled and fluctuated within the 504–650 μmol mol^-1^ range owing to the plants’ respiration that releases CO_2_ (Table 1).

**Table 1:** Treatment details of experiments conducted in the Korean Soil-Plant-Atmosphere-Research (KSPAR) chamber.

Treatment	aCaT	eCaT	aCeT	eCeT
Temperature (°C)				
Daily mean, day	20.9 ± 0.18	20.9 ± 0.18	24.9 ± 0.18	24.9 ± 0.18
Daily mean, night	14.6 ± 0.15	14.6 ± 0.15	18.6 ± 0.15	18.6 ± 0.15
CO_2_ (μmol mol^-1^) concentration				
Daily mean (day/night)	417/504	771/508	425/518	804/650
Soil temperature (°C)				
Daily mean	16.4 ± 0.28	17.3 ± 0.30	19.4 ± 0.35	18.7 ± 0.35
Soil water content (% soil volume)				
Daily mean	36.9 ± 0.31	33.1 ± 0.44	33.2 ± 0.44	33.9 ± 0.32

aCaT: Ambient 400 μmol mol^-1^ CO_2_, ambient average temperature, eCaT: Elevated 800 μmol mol^-1^ CO_2_, ambient average temperature, aCeT: Ambient 400 μmol mol^-1^ CO_2_, ambient average-temperature increases of 4°C, eCeT: Elevated 800 μmol mol^-1^ CO_2_, ambient average-temperature increases of 4°C. Values represent means ± SE.

The temperature setting of the chamber was adjusted by the Unit of the week (once every week) based on the mean temperature data of the past 20 years (1991–2010) in Jeonju area and the daily air temperature was adjusted to follow a diurnal curve pattern similar to the natural environment; the error range among the chambers was ± 0.3°C (Table 1 and S1 Fig). The daily mean solar radiation during the period of cultivation was 9.97 MJ m^-2^ (S2 Fig).

### Plant materials and growth conditions

The potato (*Solanum tuberosum* L. cv. Superior) cultivar is widely cultivated in Korea. Seed potatoes of this cultivar were obtained from the Highland Agriculture Research Institute (Pyeongchang, Korea) and stored at 10–12°C.

The quantity of chemical fertilizer was based on the topdressing by N-P_2_O_5_-K_2_O (54-30-42 g/chamber) before seeding. The soil composition in the soil bin was sandy loam (72.8% sand, 22.9% silt, and 4.3% clay). On March 23, 2018, 30 sprouted tubers were planted into each chamber with the row space of 20 cm and the plant space of 15 cm, at ~10 cm depth, and the soil moisture content was maintained at ≥ 30% (v/v) using the micro-tube-pipe of pressure-compensated drippers for the watering at 08:00 and 17:00.

### Plant measurements

Plant height, number of branches, and biomass were measured for six plants from each chamber at 35 days after emergence (DAE), a mid-phase in tuber initiation, and at 58 DAE, during tuber bulking. At the same time, the leaf area was measured using a leaf-area meter (LI-3100C, LI-COR Biosciences Inc., NE, USA), and the leaf, stem, and tuber biomass was measured after drying for 4 d at 75°C.

On June 18, 2018, the remaining potatoes were harvested and divided into leaf, stem, and tuber. The potatoes were classified into size groups (< 30 g, 30–80 g, and > 80 g), and a marketable yield was calculated based on the > 80 g class. The plant materials were oven-dried for 4 d at 75°C to obtain the biomass of each plant part.

### Stomatal conductance and chlorophyll concentration

The stomatal conductance and chlorophyll concentration of five potato leaves from each chamber was measured at 34, 43, 50, 56, and 64 DAE using a portable leaf porometer (SC-1, Mater Group Inc., USA) and a chlorophyll-content meter (CCM-300, Opti-Sciences Inc., Hudson, USA), respectively, following the manufacturer’s guidelines.

### Organic carbon, nitrogen, and mineral nutrients

To measure the concentration of carbon, nitrogen, phosphorus, potassium, and magnesium, potato leaves were collected from three potato plants of each chamber at 35, 43, 50, 58, and 64 DAE, dried at 75°C for 4 d, finely ground using a stainless steel grinder, and then sieved through 2-mm mesh.

Carbon and nitrogen were measured using the Dumass method, analyzing 0.2 g of each ground-leaf sample using an elemental analyzer (Elementar, vario MAX cube, GmbH, Germany) [55]. Phosphorus, potassium, and magnesium were measured by decomposing 0.5 g ground-leaf sample in 5 ml H_2_SO_4_ and 20 ml H_2_O_2_ for 4 h at 220°C, diluting the resulting solution to 50 ml with distilled water, and then analyzing 3 ml using inductively coupled plasma-mass spectrophotometry (ICP Integra XL, GBC Scientific Equipment Pty Ltd., Braeside, Australia) [56].

### CO_2_ gas exchange

The KSPAR chamber is a semi-closed chamber that is equipped with two CO_2_ analyzers and fans. Therefore, the CO_2_ exchange rate (*CER*; mol CO_2_ m^-2^ s^-1^) was estimated using the concentrations of CO_2_ flowing in and out of the chamber and the following equation:

$$CER=V\times P\times \;(\Delta CO_{2})\;/\;(R\times T)$$ (1)

where *V* is wind velocity (m s^-1^), *P* is the absolute pressure, Δ*CO*_*2*_ is the difference in CO_2_ concentration between the inlet and outlet (ppm), *R* is the absolute atmospheric pressure (L·atm·K^-1^·mol^-1^), and *T* is the absolute temperature (273.15 K).

The net photosynthetic rate (μmol CO_2_ m^-2^ s^-1^) for the CO_2_ exchange rate was determined during the day (05:00–19:00) when CO_2_ was supplied. The night respiration rate (μmol CO_2_ m^-2^ s^-1^) was determined from 19:00–05:00. The CO_2_ exchange rates of all the chambers were collected at 30-sec intervals, and the measured data were stored as the mean of the values measured during 5 min.

The accumulation of carbon in the plant body was calculated as follows:

$$A_{T}\;=\;(\sum_{19:00}^{05:00}P_{\text{N}}\times 12)$$ (2)

where *A*_T_ is the overall accumulated C in the plant body (g C plant^-1^), *P*_N_ (mol CO_2_ m^-2^ s^-1^) is the mean per plant body for 5 min calculated by Eq (1), and 12 is the molecular weight of carbon. The actual dark respiration rate was not used.

### Statistical analysis

The data were evaluated by analysis of variance (ANOVA) performed using statistical analysis systems 9.2. The mean values of plant traits in response to applied treatments were compared using Tukey’s tests at *P* < 0.05.

## Results

### Plant growth and biomass dynamics

**Fig 2:**
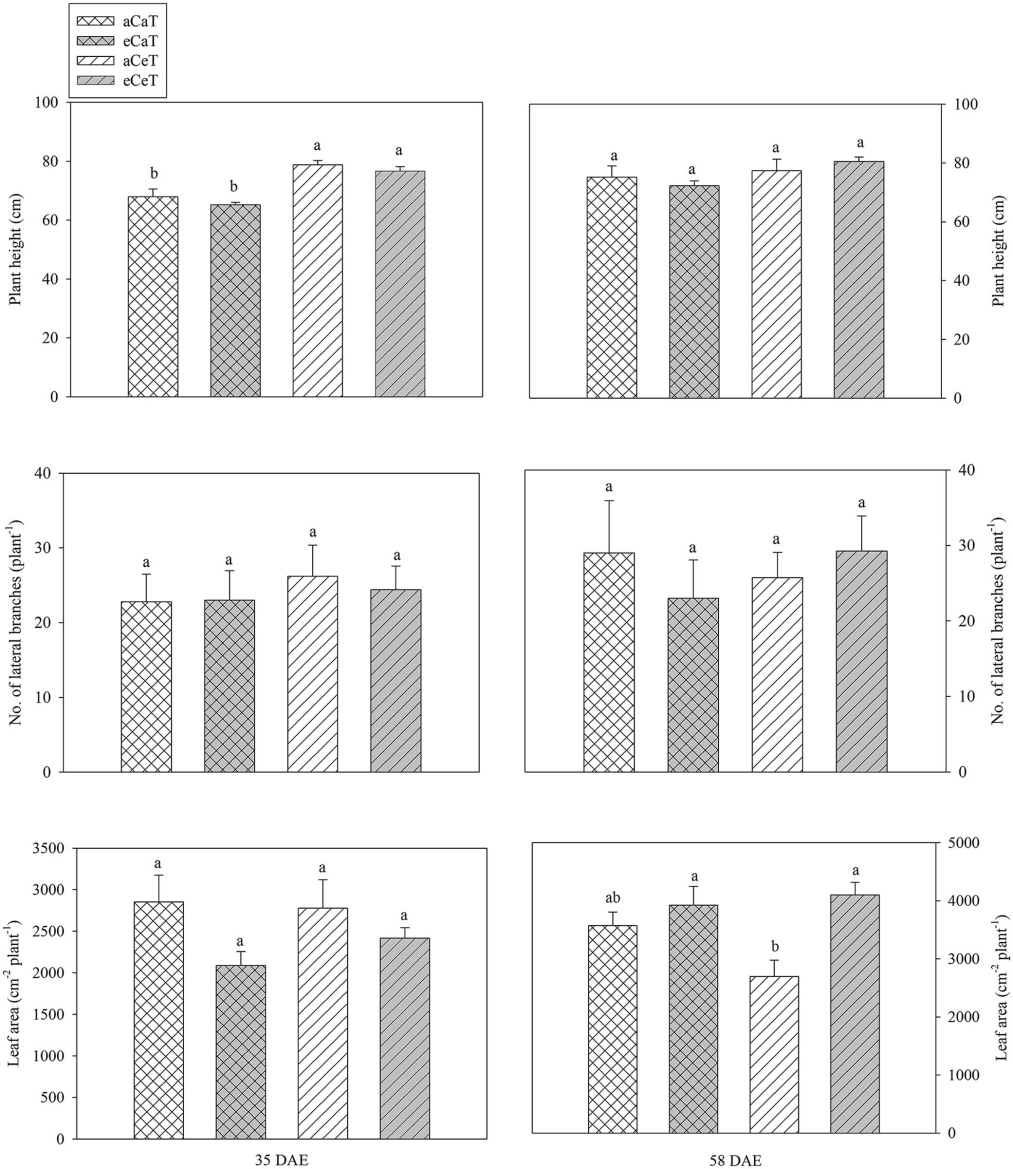
Growth parameters of potato plants at 35 DAE and 58 DAE under different conditions of CO_2_ and temperature. Values are mean ± SE (*n* = 6). Bars showing different letters indicate significant differences among treatments at *P* < 0.05 according to the Tukey test. DAE: Days after emergence.

To examine the effect of the CO_2_ concentration and temperature elevation on the growth of potato plants, the plant height, number of branches, and leaf area measured on 35 and 58 DAE were analyzed (Fig 2 and S1 Table).

At 35 DAE, plant height was significantly higher under the aCeT and eCeT conditions compared to aCaT and eCaT conditions (*P* < 0.001), whereas neither the number of branches nor leaf area was significantly affected. At 58 DAE, neither plant height nor the number of branches was significantly affected, whereas the leaf area was drastically reduced under the aCeT conditions.

**Fig 3:**
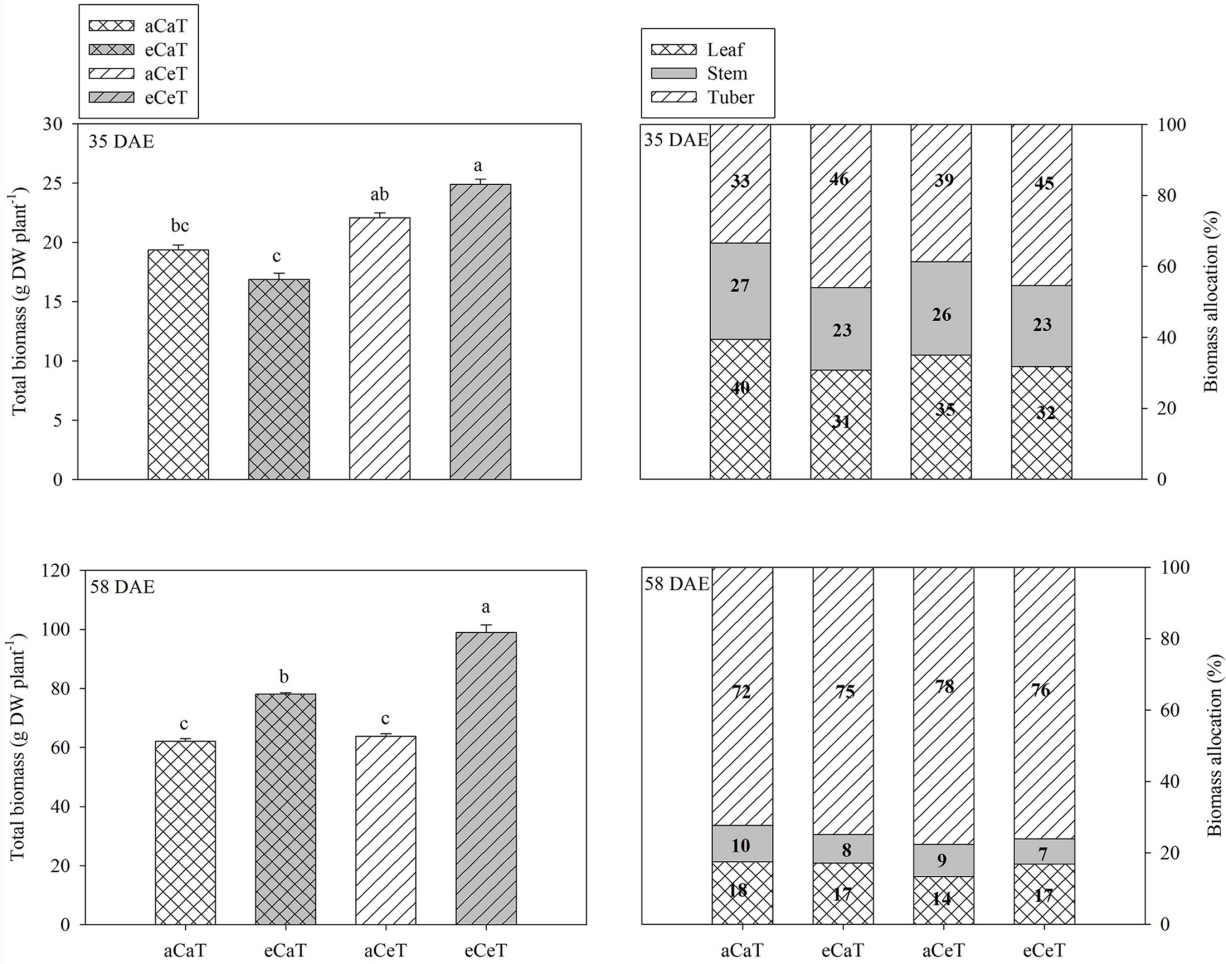
Total biomass and percent allocation of biomass in different plant parts of potato plants at 35 DAE and 58 DAE under different conditions of CO_2_ and temperature. Values are mean ± SE (*n* = 6). Bars showing different letters indicate significant differences among treatments at *P* < 0.05 according to Tukey’s test. DAE: Days after emergence.

To examine the growth characteristics of each organ relative to the potato leaf, stem, and tuber, the total biomass and the percent allocations of biomass were determined (Fig 3 and S1 Table).

At 35 DAE, total biomass was significantly higher under the aCeT and eCeT conditions than under the aCaT and eCaT conditions (*P* < 0.001). The proportion of biomass allocation to leaves and stems was lower under eCaT and eCeT conditions than under the other conditions whereas the proportion of biomass allocation to tubers was high. At 58 DAE, the total biomass was significantly higher under the eCaT and eCeT conditions, and the total biomass of plants grown under the eCaT and eCeT conditions was significantly higher by 25.6% and 59.2%, respectively, compared to the aCaT condition (*P* < 0.001). The proportion of biomass allocation was the lowest for the leaves of plants grown under aCeT condition due to the deviation from the optimal growth-temperature range during the late growth phase.

### Canopy net photosynthesis and carbon balance

**Fig 4:**
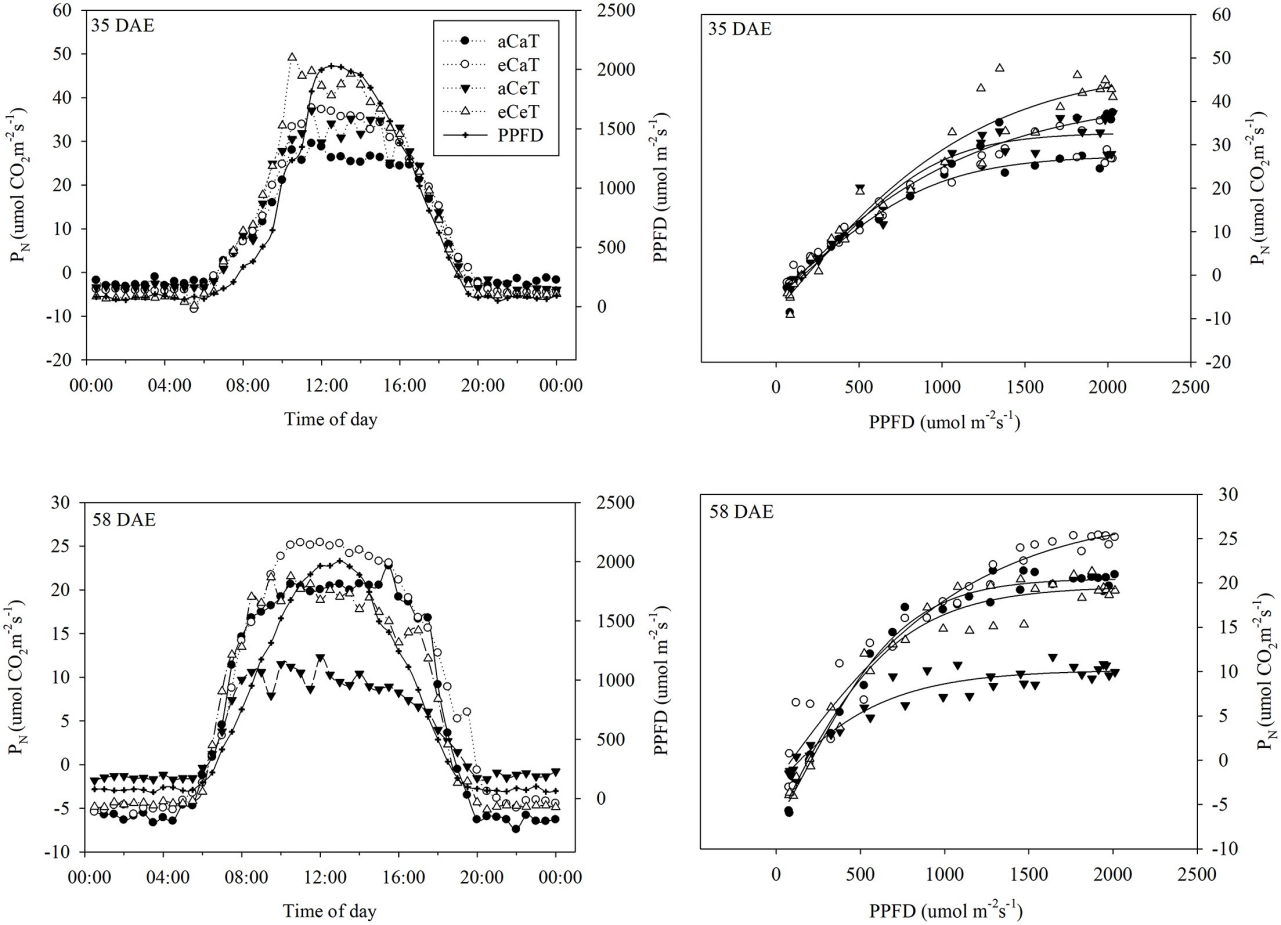
The diurnal net photosynthetic rate (P_*N*_) and Photosynthetic Photon Flux Density (PPFD) of potato plants at 35 DAE and 58 DAE under different conditions of CO_2_ and temperature.

Both CO_2_ and temperature affected canopy net photosynthetic rates between 35 DAE and 58 DAE (Fig 4).

**Fig 5:**
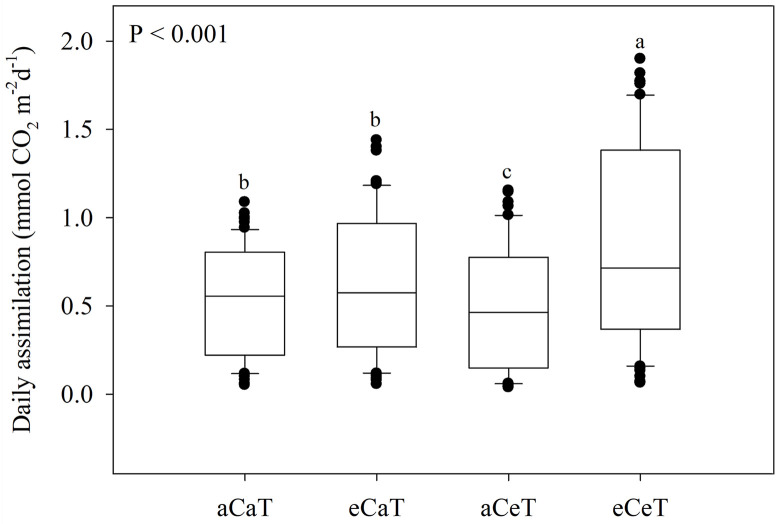
Comparison of daily canopy net photosynthesis levels from 25 DAE to 64 DAE for potato plants grown under four different conditions of CO_2_ and temperature. Means labeled with different letters differ significantly at *P* < 0.05.

The canopy net photosynthetic rates at 35 and 58 DAE increased rapidly between 08:00 a.m. and 11:00 a.m. At 35 DAE, the canopy net photosynthetic rate and light-response curve were highest under eCeT conditions followed by the aCeT, aCaT, and eCaT conditions. At 58 DAE, the canopy net photosynthetic rate and light-response curve were highest under eCaT conditions; however, there was no difference between values for plants grown under the aCaT and eCeT conditions even though lower values were observed under aCeT conditions. To compare daily canopy net photosynthesis levels between each condition of CO_2_ concentration and temperature during the growth period, the box-plot analysis was carried out (Fig 5).

**Fig 6:**
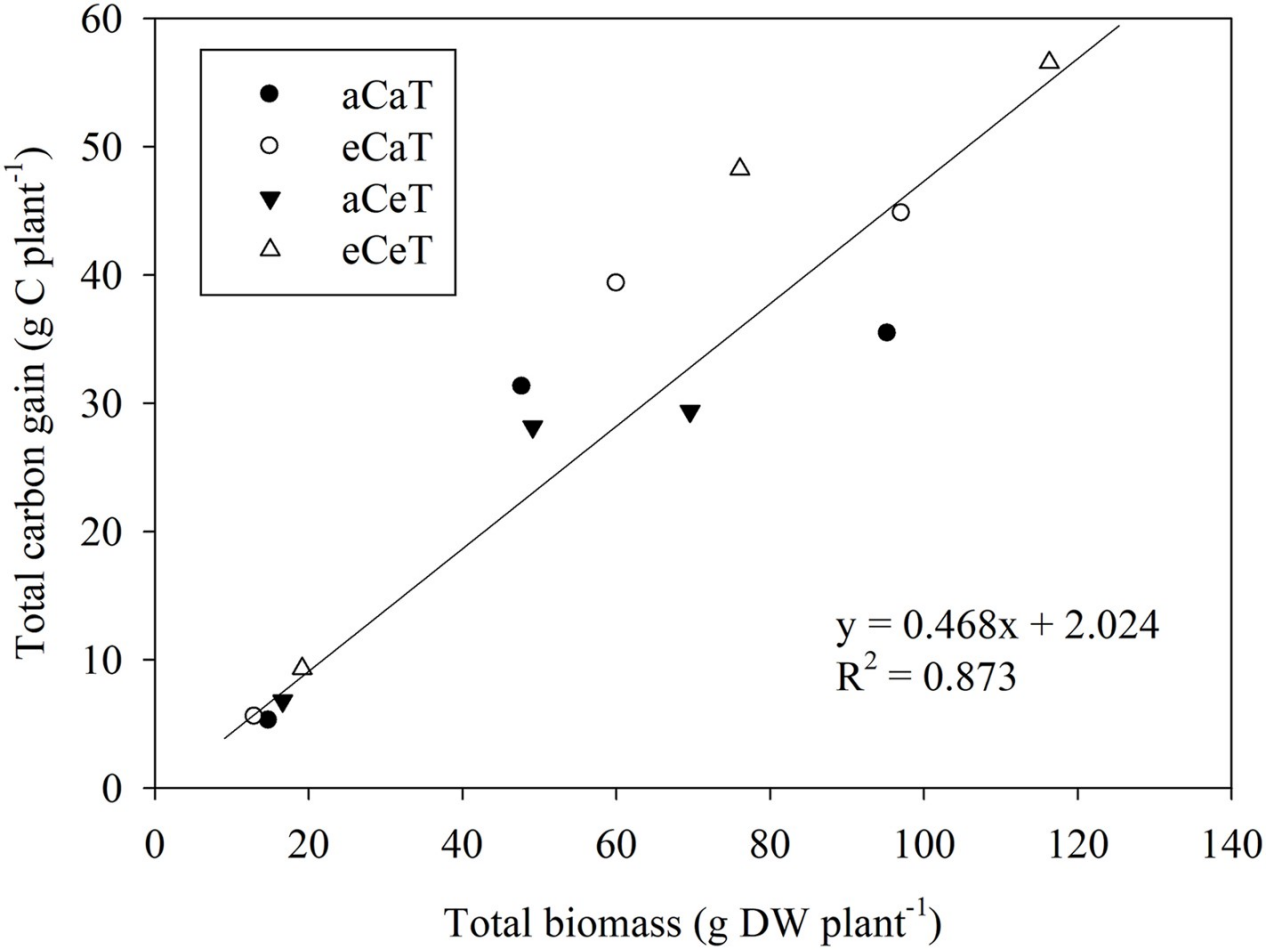
The relationship between the total carbon gain and total above-ground dry biomass of potato plants grown under different conditions of CO_2_ and temperature. Potato plants were collected at 35 DAE, 58 DAE, and the final harvest growth stages.

Net photosynthesis was significantly higher under the eCeT than under the other conditions (*P* < 0.001) and there was no significant difference in the values observed under the aCaT and eCaT conditions. However, the lowest value was obtained under aCeT conditions. The close association (r^2^ = 0.873) between total carbon gain and total dry biomass was determined using Eq (2) (Fig 6). Since the most of plant’s dry biomass (>90%) is derived from photosynthesis and the CER is closely related to plant’s dry biomass production, CER is an excellent indicator of plant growth and it has a close relationship between total carbon gain and above-ground total dry biomass [24, 34]. The slope (0.468) of the linear regression indicated that the carbon fixed from net photosynthesis accounted close to 46.8% (or 0.468 g C g^-1^ dry biomass) of the total above-ground dry biomass; in potatoes, it is generally known to be 0.4–0.46 [24, 34].

### Leaf stomatal conductance and chlorophyll concentration

**Fig 7:**
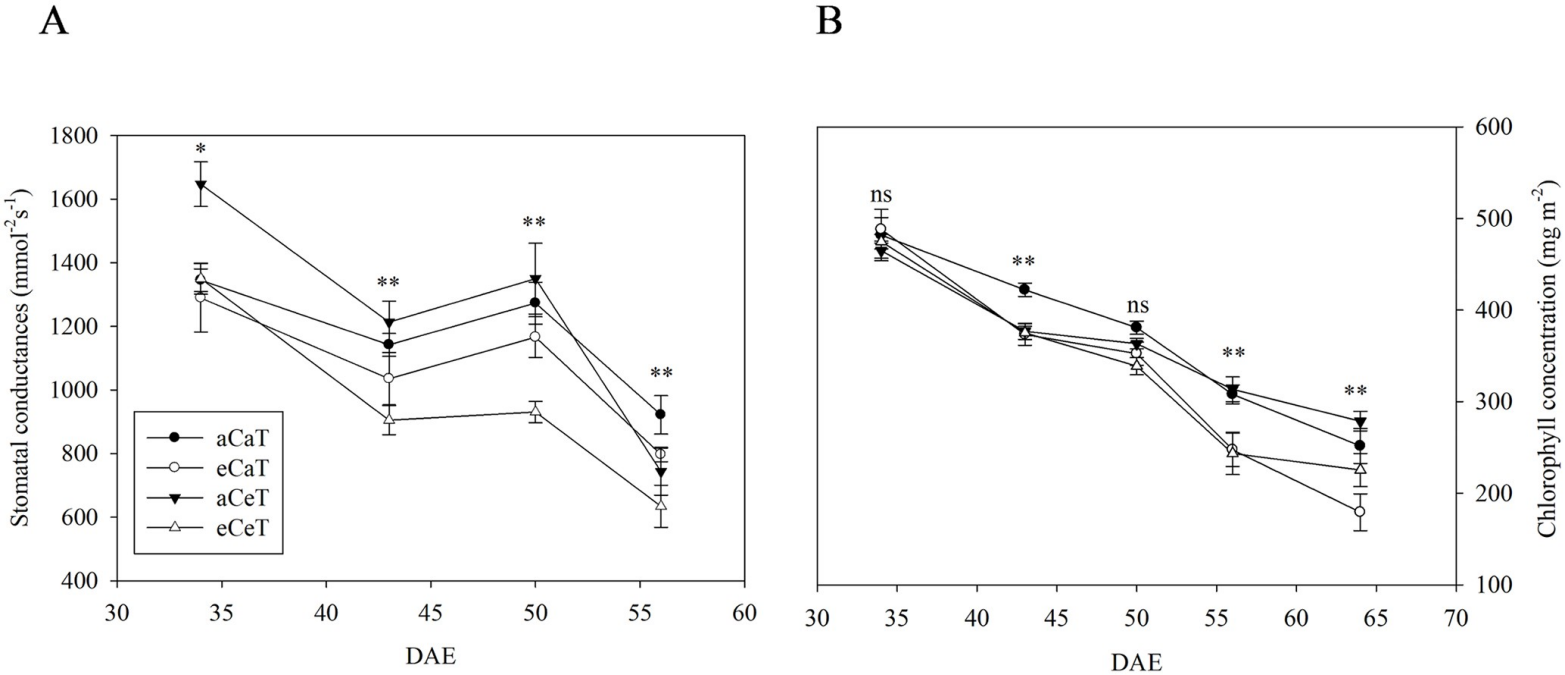
Stomatal conductance (A) and chlorophyll concentration (B) in potato leaves at 34, 43, 50, 56, and 64 DAE at different CO_2_ and temperature conditions. Bars indicate significant differences among treatments at *p* < 0.05 based on Tukey’s test. DAE: Days after emergence.

The stomatal conductance and chlorophyll concentration changed significantly during the growth period depending on CO_2_ concentration and temperature (Fig 7).

Stomatal conductance on 34 DAE was higher under aCeT conditions than under the other conditions (Fig 7A). From 42 DAE, an average reduction of 15% in stomatal conductance was observed under the eCaT and eCeT compared to the aCeT and aCaT conditions. A sharp decline in stomatal conductance was observed under aCeT conditions during the late-growth phase. The chlorophyll concentration on 34 DAE was unaffected by either temperature or CO_2_ concentration. However, from 50 DAE, the chlorophyll concentration decreased by 9.4% under the eCaT and eCeT compared to the aCeT and aCaT conditions (Fig 7B).

### Organic carbon and mineral nutrients

**Fig 8:**
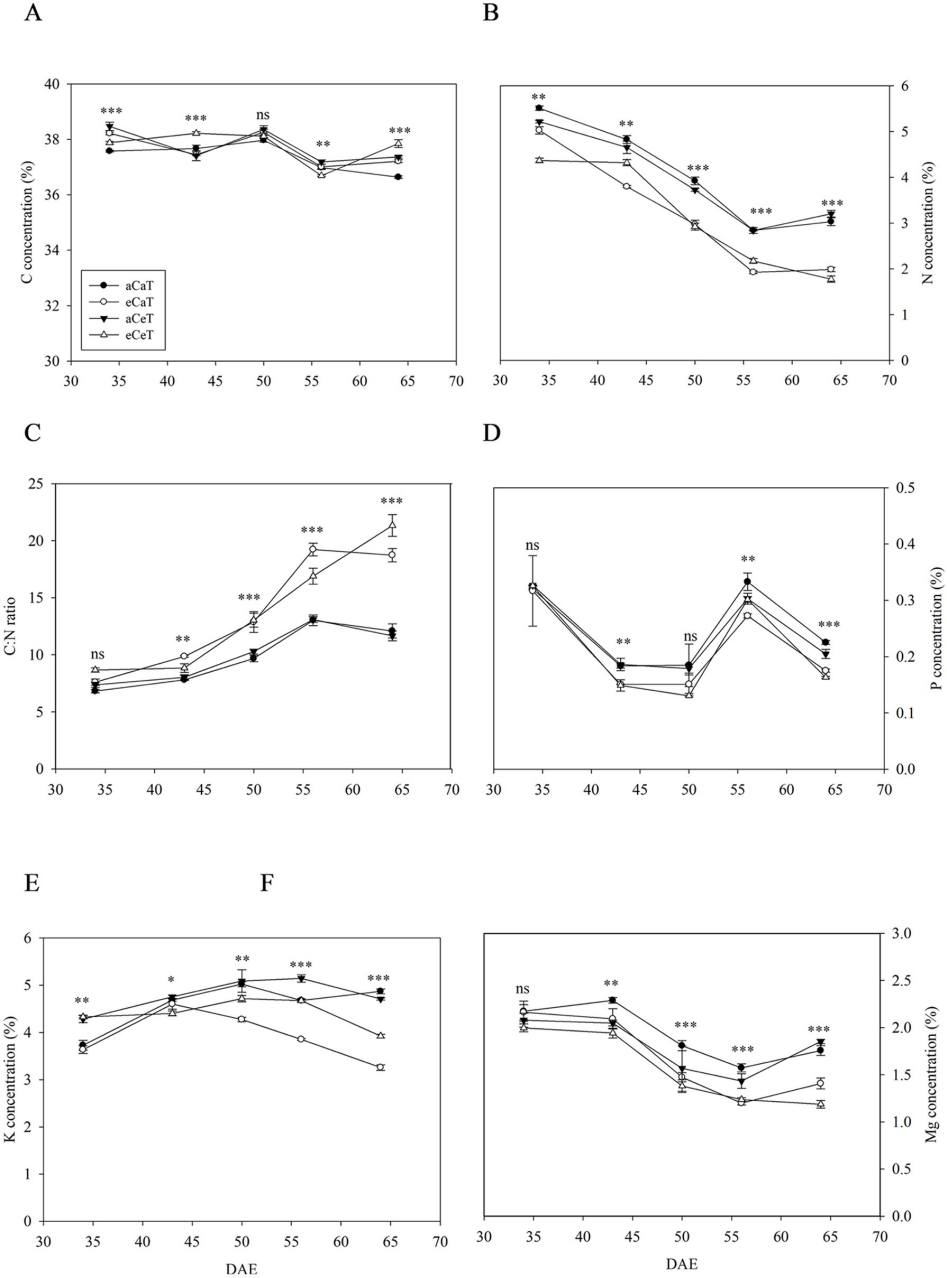
Carbon (A), nitrogen (B), C: N ratio (C), phosphorus (D), potassium (E), and magnesium (F) in potato leaves at 35, 43, 50, 58, and 64 DAE under different CO_2_ and temperature conditions. Values are mean ± SE (*n* = 3). Bars indicate significant differences among treatments at *P* < 0.05 according to Tukey’s test. DAE: Days after emergence.

The changes in the concentration of carbon, nitrogen, phosphorus, potassium, and magnesium in leaves according to the CO_2_ concentration and temperature during the growth period were analyzed (Fig 8).

Carbon concentrations showed statistically significant differences depending on the growth stage and treatment conditions but not as much as nitrogen concentrations (Fig 8A). The nitrogen concentrations were on average 24% lower under the eCaT and eCeT compared to the aCeT and aCaT conditions (Fig 8B). These differences were also reflected in the C:N ratio (Fig 8C). The concentrations of phosphorus, potassium, and magnesium were generally lower (16% on average) under the eCaT and eCeT compared to the aCeT and aCaT conditions (Fig 8D–8F and S2 Table).

### Tuber yield and biomass

At harvest time, the tuber size, biomass, yield, and marketable yield of potatoes cultivated under varying CO_2_ concentrations and temperatures were analyzed (Table 2).

**Table 2:** Numbers of different sized and total tubers, biomass, tuber yield, and harvest index of potato plants under different treatment conditions of CO_2_ and temperature at the final harvest.

Treatment	No. tubers (plant^-1^)			Total no. tubers (plant-1)	Biomass (g DW plant^-1^)			Yield (g FW plant^-1^)		Harvest Index\
	<30 g	30–80 g	>80 g		Leaf	Stem	Tuber	Tuber	Marketable	
aCaT	4.8^a^	2.9^b^	2.3^b^	10.2^a^	10.4^b^	7.1^b^	106.4^b^	502^b^	273^b^	0.87^ab^
eCaT	5.2^a^	3.9^a^	2.4^b^	11.5^a^	9.8^b^	6.2^b^	110.3^b^	527^b^	269^b^	0.89^a^
aCeT	2.0^c^	2.8^b^	1.9^c^	6.4^b^	7.6^b^	6.0^b^	76.9^c^	342^c^	195^c^	0.84^ab^
eCeT	3.9^b^	4.1^a^	2.9^a^	10.8^a^	14.4^a^	9.3^a^	127.5^a^	604^a^	352^a^	0.82^b^
ANOVA										
CO_2_	***	**	***	***	***	*	***	***	***	ns
Temp	***	ns	Ns	**	**	Ns	**	*	Ns	**
CO_2_ × Temp	***	ns	***	**	***	***	***	***	***	ns

Means followed by the same letters in each column are not significantly different at *P* < 0.05 according to Tukey’s test.

*Significant at the 0.05 probability level;

** Significant at the 0.01 probability level;

***Significant at the 0.001 probability level;

NS—not significant; Tuber size: <30 g Small, 30–80 g Medium, >80 g Large; Marketable: weight > 80 g; Harvest index: tuber dry weight / total dry weight; Temp: Temperature.

There were significant differences in the number of small (< 30 g), medium (30–80 g), and large (> 80 g) potatoes. The tuber size was greater under eCeT than under the eCaT, eCaT, and aCeT conditions and was the smallest under aCeT conditions. There was no significant difference in the total number of tubers per plant among the aCaT, eCaT, and eCeT conditions but was lower at the aCeT conditions.

Biomass was significantly affected by the treatment conditions, with the highest leaf, stem, and tuber biomass achieved under eCeT conditions. There was no significant difference in the biomass observed under the aCaT and eCaT conditions. The biomass was much lower under aCeT conditions.

Tuber yield and marketable yield were significantly affected by the treatment conditions and were 604 and 352 g, respectively, under eCeT conditions. Similar to the observed biomass, there was no significant difference in either tuber yield or marketable yield under the aCaT and eCaT conditions. Both the tuber and marketable yields were lower under the aCeT conditions (342 and 195 g, respectively).

The harvest index varied significantly across the treatment conditions, with the greatest index observed under eCaT conditions. ANOVA indicated that tuber yield was significantly affected by both the CO_2_ concentration and the CO_2_ × temperature interaction, whereas the harvest index was affected only by temperature.

## Discussion

### The main effects of elevated temperature

During the early growth phase, plant height, biomass, and canopy net photosynthetic rate were higher under the aCeT and eCeT conditions (Figs 2, 3, and 4). Under the aCeT condition, however, leaf stomatal conductance, canopy net photosynthetic rate, and leaf area all declined sharply as the conditions deviated from the optimal growth temperature at the late-growth phase. As a result, the biomass and yield of the harvested tubers were significantly lower than those obtained under the other treatment conditions.

During the early growth phase of potato, the elevated temperature has been reported to facilitate the development of the above-ground portion of the plant [13, 14, 24]. However, when the air temperature deviates from the effective range, the net photosynthetic rate falls at both the leaf and canopy levels and limits the accumulation of assimilates [22, 23, 57]. The activity of PS II, where the photosynthetic reactions occur, has been reported to increase within the effective temperature range for potato (18–20°C) but to decrease at higher temperatures [17, 23, 24, 58, 59]. As the leaf temperature rises, the photorespiration rate also rises faster than the photosynthesis rate because under high-temperature conditions the specificity of the Rubisco enzyme for CO_2_ decreases compared to the specificity for O_2_ and the solubility of O_2_ decreases less than that of CO_2_ making it a more favorable environment for oxygenation reactions [49]. The accelerated loss of assimilated CO_2_ consequently reduces the photosynthetic rate [48, 60] and ultimately leads to a decrease in the weight and yield of the tubers [12, 15, 19, 24].

#### The main effects of increased CO_2_ concentration

In the present study, potato plants grown under the elevated CO_2_ condition showed a relatively lower leaf area and biomass at the early growth stage than the other conditions. This outcome partially agrees with the results of previous OTC and FACE studies that reported that elevated CO_2_ concentrations could reduce the above-ground growth during the early growth phase [30, 50–52]. Towards the late-growth phase, the canopy net photosynthetic rate and biomass were relatively higher under the eCaT than under the aCaT conditions but the final slightly increased tuber yield was not statistically different from that obtained under the aCaT conditions. Previous studies conducted in a growth chamber, OTC, and FACE have demonstrated that although the effect varies depending on the experimental conditions, elevated CO_2_ concentrations have beneficial effects on the photosynthetic rate, growth, and yield of potatoes [27, 28, 30, 50–52, 61]. However, in this study, the eCaT condition with only an elevated CO_2_ did not lead to a distinct increase in the yield of potatoes; this outcome may be related to the photosynthesis acclimation observed upon long-term exposure to elevated CO_2_ concentrations or the relatively densely grown environments.

Studies have reported that stomatal conductance and chlorophyll concentrations decrease at CO_2_ concentrations two-times higher than the current atmospheric levels [11, 62–65]. The findings of the present study concur that both stomatal conductance and chlorophyll concentrations decrease rapidly towards the late-growth phase when plants are grown under elevated CO_2_ concentrations (Fig 7B). Stomatal conductance is known to decrease under elevated CO_2_ conditions, and the responses vary according to the diversity of plant species and the environment [7]. A particularly steep decrease occurs upon long-term rather than short-term exposure [39, 45]. On the other hand, long-term exposure to elevated CO_2_ concentrations causes photosynthesis acclimation that is accompanied by a reduced stomatal conductance, a decrease in leaf chlorophyll concentration, and a reduction in the Rubisco activation state in the photosynthetically active source leaves [36].

### The interaction of temperature and CO_2_

Many of the effects of elevated CO_2_ on plant growth and carbon metabolism are offset or influenced by elevated temperatures [49]. For example, the inhibition of photorespiration by elevated CO_2_ is temperature-dependent and the benefits of increased CO_2_ in photosynthesis are greater at higher temperatures. To realistically analyze how climate change affects crops, these climate-change factors need to be investigated together. A recent study in rice has shown that the interactive effects of elevated CO_2_ and temperature are advantageous for photosynthesis but not for the growth and yield of rice [4]. It was found that the high-temperature condition causes yield loss regardless of whether it is a high temperature alone or combined with an elevated CO_2_ concentration.

In the present study, potato plants grown under eCeT conditions exhibited the greatest height, leaf area, total biomass, and canopy net photosynthetic rates during the early growth phase. Towards the late-growth phase, the canopy net photosynthetic rate became lower than that of plants grown under eCaT conditions (Fig 4). As a result, during the early growth phase, the photosynthetic rate was high under the eCeT condition owing to the elevated temperature. However, during the late growth phase, the elevated CO_2_ and temperature are known to affect the assimilates and cause them to accumulate in mesophyll cells or chlorophylls, thereby reducing the photosynthetic rate [11, 62]. Generally, the net photosynthetic rate decreases more rapidly than the respiration rate when the temperature is elevated and the net assimilation has been reported to decrease above certain temperature levels [63, 66]. Nevertheless, under the eCeT condition, the biomass, yield, and marketable yield of potato tuber was the highest among the treatment conditions at the final harvest because starch, as an assimilate, was accumulated in the tubers for storage. Correspondingly, the accumulated ratio of assimilates produced by the net photosynthesis under the elevated CO_2_ concentrations is relatively higher, whereby the proportion of tubers in the total biomass increases [27]. The high tuber yield in this study was attributed to the increase in tuber size rather than in tuber number. The findings agree with previous studies showing that the increase in tuber yield under elevated CO_2_ conditions is mainly due to an increase in the size of already initiated tubers rather than an increase in the number of tubers [27, 30, 47, 50, 67].

Elevated CO_2_ concentrations can help restore PS II activity after it is reduced by an elevated temperature [66, 68]. Even though PS II activity was not measured in the present study, we found that the concurrent elevation of temperature and CO_2_ enhanced plant thermostability and reduced the damaging effect of elevated temperature.

### Changes in organic carbon, nitrogen, and mineral concentrations

The balance between carbon and nitrogen is a critical factor in plant growth and development; in general, the concentrations of carbon and nitrogen in the plant biomass are 45% and 5%, respectively [69]. In the present study, the elevation of CO_2_ concentration had negative effects on the concentrations of nitrogen, as well as phosphorus, potassium, and magnesium (Fig 8); this is consistent with the previous FACE, OTC, CTC, glasshouse, and growth-chamber studies showing that the elevation of CO_2_ concentration can reduce nitrogen concentration by 7–18% depending on the crop species and variety [41, 70–73]. However, the decrease observed in potatoes was relatively large compared to the losses observed in other crop species.

The photosynthesis of C_3_ plants is mediated *via* CO_2_ fixation in Calvin’s cycle and CO_2_ is fixed by the enzyme Rubisco that catalyzes the carboxylation of ribulose-1,5-bisphosphate, the first major step of carbon fixation. Some studies have reported that prolonged exposure to elevated CO_2_ lowers the content and activity of Rubisco enzyme and causes an increase in the C:N ratio with lower N levels resulting in lower protein levels [26, 36, 41]. Increased levels of assimilates resulting from the elevated CO_2_ concentration have been reported to dilute the nitrogen concentration of plants [72].

Overall, elevated CO_2_ conditions reduce stomatal conductance, inhibit the rubisco-activating enzyme, and increase the C:N ratio. However, the efficiency of nitrogen use has been reported to increase despite reductions in nitrogen concentration [35]. The level of magnesium contained in chlorophyll has been reported to decrease by 1–8% under elevated CO_2_ conditions [40, 74]. Elevated CO_2_ can cause the accumulation of assimilates in photosynthetically active source leaves when the photosynthetic rate exceeds the export capacity or the capacity of sink organs, thereby reducing the photosynthetic rate [11, 62]. Thus, source-sink imbalance may occur under the elevated CO_2_ condition and the delayed transport or accumulation of assimilates may drive the reduction in photosynthetic rate during the late-growth phase.

## Conclusions

The study examined the effects of CO_2_ concentration and temperature on the growth, yield, and photosynthesis of potato crops using a KSPAR chamber. At elevated temperature, both stomatal conductance and canopy net photosynthetic rate decreased sharply; as the temperature deviated from the effective range towards the late-growth phase, both biomass and tuber yield were reduced. At an elevated CO_2_ level, the area and biomass of leaves that create photosynthetic products increased during the late-growth phase. However, the tuber yield did not change significantly compared to that obtained under the aCaT conditions. The concurrent elevation of CO_2_ and temperature caused a distinct difference in the growth, development, and canopy net photosynthetic rate of potato plants compared to independent elevation. This increased biomass and tuber yield was due to the enlarged tuber size rather than to an increase in the number of tubers.

The source and sink organs of crop plants can change according to the external environment and the influence of the CO_2_ and temperature is particularly significant. Our study indicates that excessive increases in only one of these parameters will prevent the balanced development of the source and sink organs of potatoes. However, in conditions of concurrently elevated CO_2_ and temperature, the source and sink organs are likely to exhibit balanced development that may have positive effects on crop growth and quality. The elevation of CO_2_ and temperature within the effective range will exert positive effects on the growth, yield, and photosynthesis of potato crops. To elucidate the conditions of concurrent elevation in a more systematic way, molecular-biology experiments should be conducted.

## Supporting information

S1 Fig Temperature setting for KSPAR chambers programmed to maintain ambient Temperature (aT) or ambient temperature plus 4°C (eT) conditions. DAE: days after emergence; Average: daily mean temperature of the Jeonju area (1991–2010).
(TIF)

S2 Fig Mean daily solar radiation for the Days After Emergence (DAE) at the National Institute of Crop Science (35°84'34" N, 127°04'84" E). (TIF)

S1 Table ANOVA results for the growth parameters of the potato plants grown under the four different treatment conditions. (DOCX)

S2 Table ANOVA results of C, N, C: N, P, K, and Mg parameters at 35, 43, 50, 58, 64 Days After Emergence (DAE). (DOCX)
