# Supplementary figures and images for: The effect of concurrent elevation in CO2 and temperature on the growth, photosynthesis, and yield of potato crops
Source: PLoS One. 2020 Oct 21;15(10):e0241081. doi: 10.1371/journal.pone.0241081 (PMC7577495; doi:10.1371/journal.pone.0241081)

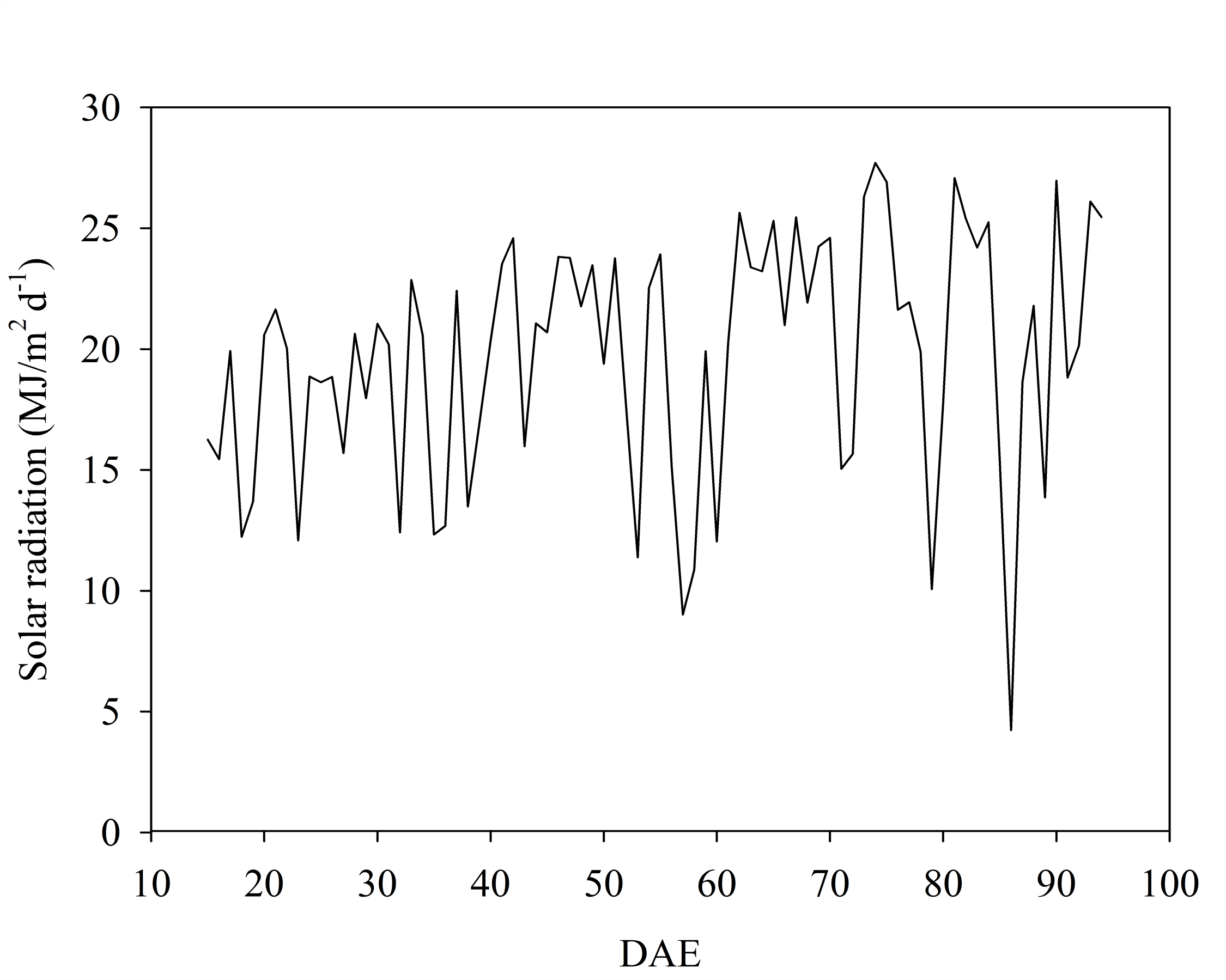

Supplement: S2 Fig — (TIF) [file pone.0241081.s002.tif]
