# Supplementary material for: The effect of concurrent elevation in CO2 and temperature on the growth, photosynthesis, and yield of potato crops
Source: PLoS One. 2020 Oct 21;15(10):e0241081. doi: 10.1371/journal.pone.0241081 (PMC7577495; doi:10.1371/journal.pone.0241081)
Supplement: S1 Table — (DOCX) [file pone.0241081.s003.docx]

**S1 Table. ANOVA results for the growth parameters of the potato plants grown under the four different treatment conditions.**

| Parameters | 34 DAE | | |  | 56 DAE | | |
| --- | --- | --- | --- | --- | --- | --- | --- |
|  | CO_2_ | Temp | CO_2_ × Temp |  | CO_2_ | Temp | CO_2_ × Temp |
| Plant height (cm) | 0.1756 | **<0.0001** | 0.8597 |  | 0.9849 | 0.2027 | 0.4504 |
| No. of lateral branches (plant^-1^) | 0.8342 | 0.5232 | 0.7937 |  | 0.8149 | 0.7789 | 0.3810 |
| Leaf area (cm^2^ plant^-1^) | 0.0202 | 0.3919 | 0.2567 |  | 0.0065 | 0.2153 | 0.0726 |
| Total biomass  (g DW plant^-1^) | 0.8688 | **<0.0001** | 0.0187 |  | **<0.0001** | 0.0158 | 0.0340 |

Means followed by the same letters in each column are not significantly different at *P* < 0.05, DAE: Days after emergence, Temp: Temperature
