# Supplementary material for: The effect of concurrent elevation in CO2 and temperature on the growth, photosynthesis, and yield of potato crops
Source: PLoS One. 2020 Oct 21;15(10):e0241081. doi: 10.1371/journal.pone.0241081 (PMC7577495; doi:10.1371/journal.pone.0241081)
Supplement: S2 Table — (DOCX) [file pone.0241081.s004.docx]

**S2 Table. ANOVA results of C, N, C: N, P, K, and Mg parameters at 35, 43, 50, 58, 64 days after emergence (DAE).**

| Concentration (%) | DAE | aCaT | eCaT | aCeT | eCeT | CO_2_ | Temp | CO_2_ × Temp |
| --- | --- | --- | --- | --- | --- | --- | --- | --- |
| C | 35 | 38.2^ab^ | 37.6^c^ | 38.5^a^ | 37.9^bc^ | 0.0112 | <0.0001 | 0.7695 |
|  | 43 | 37.4^b^ | 37.7^b^ | 37.4^b^ | 38.2^a^ | 0.0549 | 0.0020 | 0.0340 |
|  | 50 | 38.3^a^ | 38.0^a^ | 38.4^a^ | 38.1^a^ | 0.2219 | 0.0230 | 0.8140 |
|  | 58 | 37.0^ab^ | 37.0^ab^ | 37.2^a^ | 36.7^b^ | 0.4126 | 0.0047 | 0.0085 |
|  | 64 | 37.2^b^ | 36.6^c^ | 37.4^b^ | 37.8^a^ | <0.0001 | 0.5845 | 0.0002 |
|  |  |  |  |  |  |  |  |  |
| N | 35 | 5.51^a^ | 5.03^c^ | 5.22^b^ | 4.37^d^ | <0.0001 | <0.0001 | <0.0001 |
|  | 43 | 4.83^a^ | 3.80^d^ | 4.66^b^ | 4.32^c^ | <0.0001 | <0.0001 | <0.0001 |
|  | 50 | 3.92^a^ | 2.97^c^ | 3.73^b^ | 2.92^c^ | <0.0001 | <0.0001 | 0.0008 |
|  | 58 | 2.84^a^ | 1.92^c^ | 2.84^a^ | 2.17^b^ | <0.0001 | <0.0001 | <0.0001 |
|  | 64 | 3.03^b^ | 1.99^c^ | 3.20^a^ | 1.77^d^ | <0.0001 | <0.0001 | <0.0001 |
|  |  |  |  |  |  |  |  |  |
| C:N | 35 | 6.8^d^ | 7.6^c^ | 7.4^b^ | 8.7^a^ | 0.1894 | <0.0001 | <0.0001 |
|  | 43 | 7.8^d^ | 9.9^a^ | 8.0^c^ | 8.8^b^ | <0.0001 | <0.0001 | <0.0001 |
|  | 50 | 9.7^c^ | 12.9^a^ | 10.3^b^ | 13.0^a^ | 0.0001 | <0.0001 | 0.0047 |
|  | 58 | 13.0^c^ | 19.2^c^ | 13.1^a^ | 16.9^b^ | <0.0001 | <0.0001 | <0.0001 |
|  | 64 | 12.1^c^ | 18.7^d^ | 11.7^b^ | 21.3^a^ | <0.0001 | <0.0001 | <0.0001 |
|  |  |  |  |  |  |  |  |  |
| P | 35 | 0.32^a^ | 0.32^a^ | 0.33^a^ | 0.33^a^ | 0.7455 | 0.9829 | 0.9544 |
|  | 43 | 0.18^ab^ | 0.15^b^ | 0.19^a^ | 0.15^b^ | 0.9748 | 0.0020 | 0.7792 |
|  | 50 | 0.18^a^ | 0.15^a^ | 0.18^a^ | 0.13^a^ | 0.5412 | 0.0858 | 0.7324 |
|  | 58 | 0.33^a^ | 0.27^b^ | 0.29^b^ | 0.30^b^ | 0.3826 | 0.0298 | 0.0029 |
|  | 64 | 0.23^a^ | 0.18^b^ | 0.20^a^ | 0.16^b^ | 0.0097 | <0.0001 | 0.4102 |
|  |  |  |  |  |  |  |  |  |
| K | 35 | 3.73^b^ | 3.63^b^ | 4.28^a^ | 4.33^a^ | <0.0001 | 0.7670 | 0.3961 |
|  | 43 | 4.69^ab^ | 4.60^ab^ | 4.75^a^ | 4.40^b^ | 0.3441 | 0.0089 | 0.0698 |
|  | 50 | 5.02^a^ | 4.27^b^ | 5.09^a^ | 4.71^ab^ | 0.0867 | 0.0025 | 0.1848 |
|  | 58 | 4.68^b^ | 3.85^c^ | 5.14^a^ | 4.67^b^ | <0.0001 | <0.0001 | 0.0054 |
|  | 64 | 4.87^a^ | 3.26^b^ | 4.71^a^ | 3.92^c^ | 0.0005 | <0.0001 | <0.0001 |
|  |  |  |  |  |  |  |  |  |
| Mg | 35 | 2.17^a^ | 2.16^a^ | 2.08^a^ | 2.00^a^ | 0.2545 | 0.6750 | 0.7456 |
|  | 43 | 2.29^a^ | 2.09^b^ | 2.05^b^ | 1.94^c^ | 0.0002 | 0.0003 | 0.0866 |
|  | 50 | 1.81^a^ | 1.47^bc^ | 1.57^b^ | 1.38^c^ | 0.0002 | <0.0001 | 0.0182 |
|  | 58 | 1.57^a^ | 1.20^c^ | 1.43^b^ | 1.24^c^ | 0.0211 | <0.0001 | 0.0016 |
|  | 64 | 1.76^a^ | 1.41^b^ | 1.85^a^ | 1.19^c^ | 0.0062 | <0.0001 | <0.0001 |

Means followed by the same letters in each column are not significantly different at *P* < 0.05, DAE: Days after emergence, Temp: Temperature
